# Supplementary material for: Loss of family with sequence similarity 13, member A exacerbates pulmonary hypertension through accelerating endothelial-to-mesenchymal transition
Source: PLoS One. 2020 Feb 13;15(2):e0226049. doi: 10.1371/journal.pone.0226049 (PMC7018082; doi:10.1371/journal.pone.0226049)
Supplement: S1 Table — (PDF) [file pone.0226049.s001.pdf]

Supporting Information

S1 Table. Nucleotide sequence of primers.

Gene Name		Forward Primer	Reverse Primer
Mouse	*Acta1*	ACAGAGGCACCACTGAACCCTAAG	ACAATCTCACGCTCGGCAGTAGTC
	Fn1	TGTGACAACTGCCGTAGACC	GACCAACTGTCACCATTGAGG
	S100a4	GGAAGAAGTGAAGACTCCTCAGATGAAG	GCTCAGCTCTGTGCACATGTGCGGAAG
	Pecam1	AACGAGAGCCACAGAGACGGTGTAC	ATACGTGCACAGGACTCTCGCAATC
	Cdh5	TTGCCCAGCCCTACGAACCTAAAG	ACCACCGCCCTCCTCATCGTAAGT
	Twist1	ACGCAGTCGCTGAACGAGGCGTTCG	GCTCTGCAGGACCTGGTACAGGAAG
	Snai1	CGAGCTGCAGGATTCTA	GTGGGATGGCTGCCAGC
	Fam13a	CCGCTGCGAAGCTCACAGGAAG	TTGGTCTCCAGCGTTGCTGACATCA
Human	*ACTA1*	GTCACCCACAACGTGCCCATTTATG	GCCACGTAGCACAGCTTCTCCTTGAT
	FN1	TCGCCATCAGTAGAAGGTAGCA	TGTTATACTGAACACCAGGTTGCA
	S100A4	CCTGTCCTGCATCGCCATGATGTGT	CTTGCTCAGCATCAAGCACGTGTCTG
	PECAM1	GTGTCCTCAGCTGAGTCTCACAAAG	GGATGTGCATCTGGCCTTGCTGTCT
	CDH5	CCAAAGTGTGTGAGAACGCTGT	GCCGTGTTATCGTGATTATCCGTG
	TWIST1	TGGACAGTCTAGAGACTCTGGAGCTGG	ATGCAGAGGTGTGAGGATGGTGCCGCT
	SNAI1	GCACGGCCTAGCGAGTGGTT	GGGCTGCTGGAAGGTAAACTCTGG
	FAM13A	GAGTGCTCATGTACCCCAAGTCAGC	CGCTGTCTGGCAGATGTGGCAGAAG
	18s	GTAACCCGTTGAACCCCATT	CCATCCAATCGGTAGTAGCG
