# Supplementary material for: Loss of family with sequence similarity 13, member A exacerbates pulmonary hypertension through accelerating endothelial-to-mesenchymal transition
Source: PLoS One. 2020 Feb 13;15(2):e0226049. doi: 10.1371/journal.pone.0226049 (PMC7018082; doi:10.1371/journal.pone.0226049)
Supplement: S1 Fig — (PDF) [file pone.0226049.s002.pdf]

Original uncropped blot images

Figure 1A

FAM13A

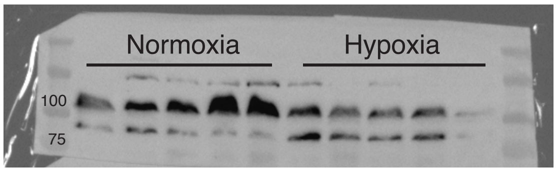

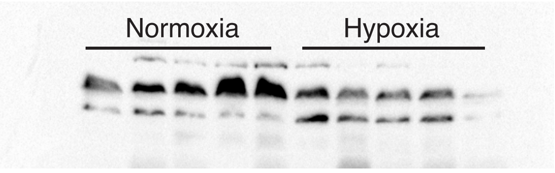

GAPDH

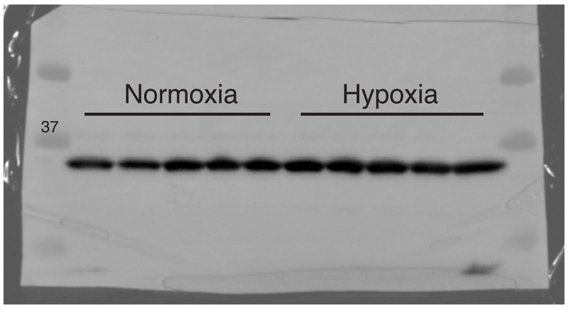

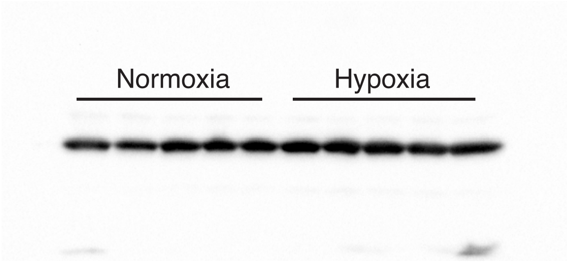

Samples: Mouse whole-lung

All blots were captured and processed using Chemi Doc XRS+ and Image Lab software

Figure 5I

FAM13A

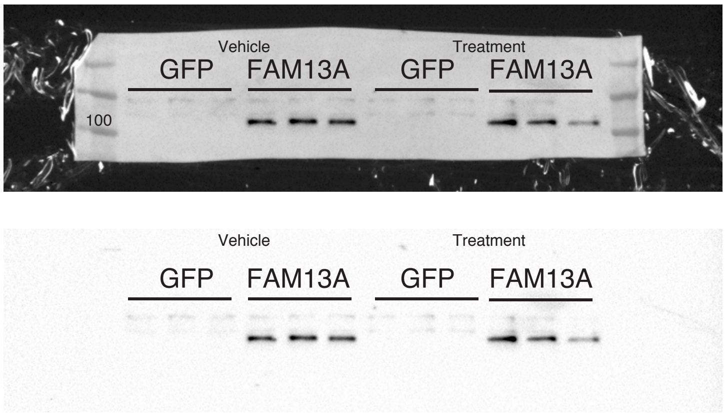

Active β-catenin

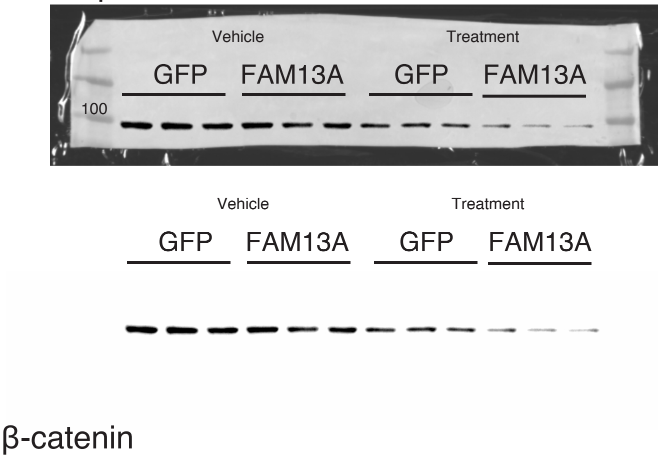

β-catenin

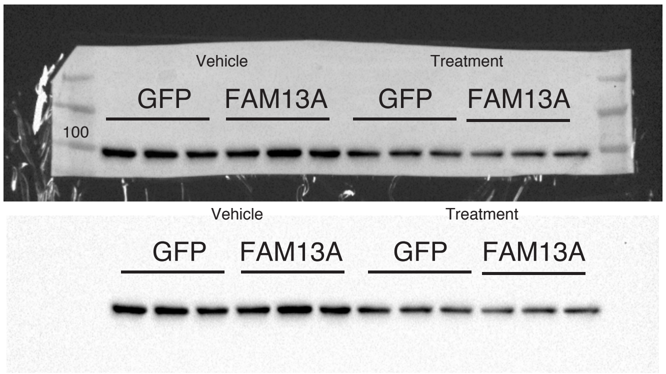

GAPDH

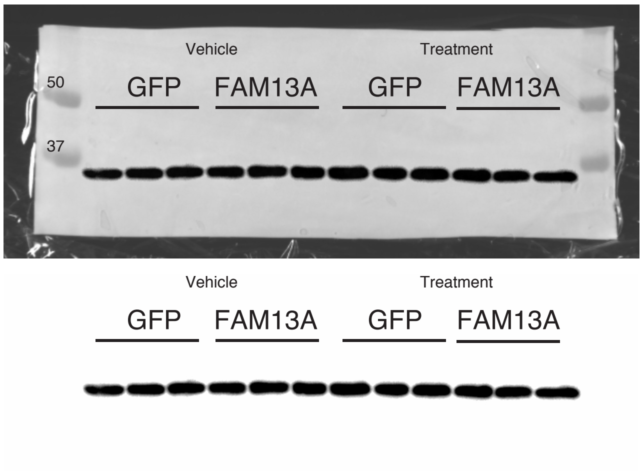

Samples: PAEC

All blots were captured and processed using Chemi Doc XRS+ and Image Lab software

Figure 5H Cleaved Caspase-3

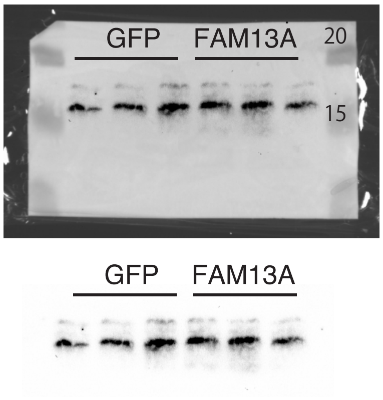

Caspase-3

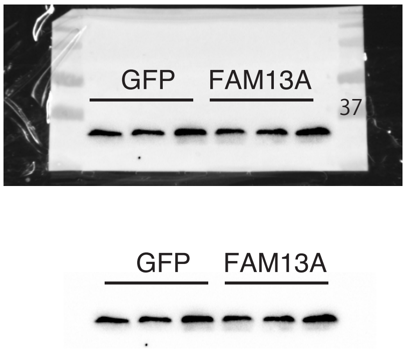

Samples: PAEC

All blots were captured and processed using Chemi Doc XRS+ and Image Lab software

Figure 4B

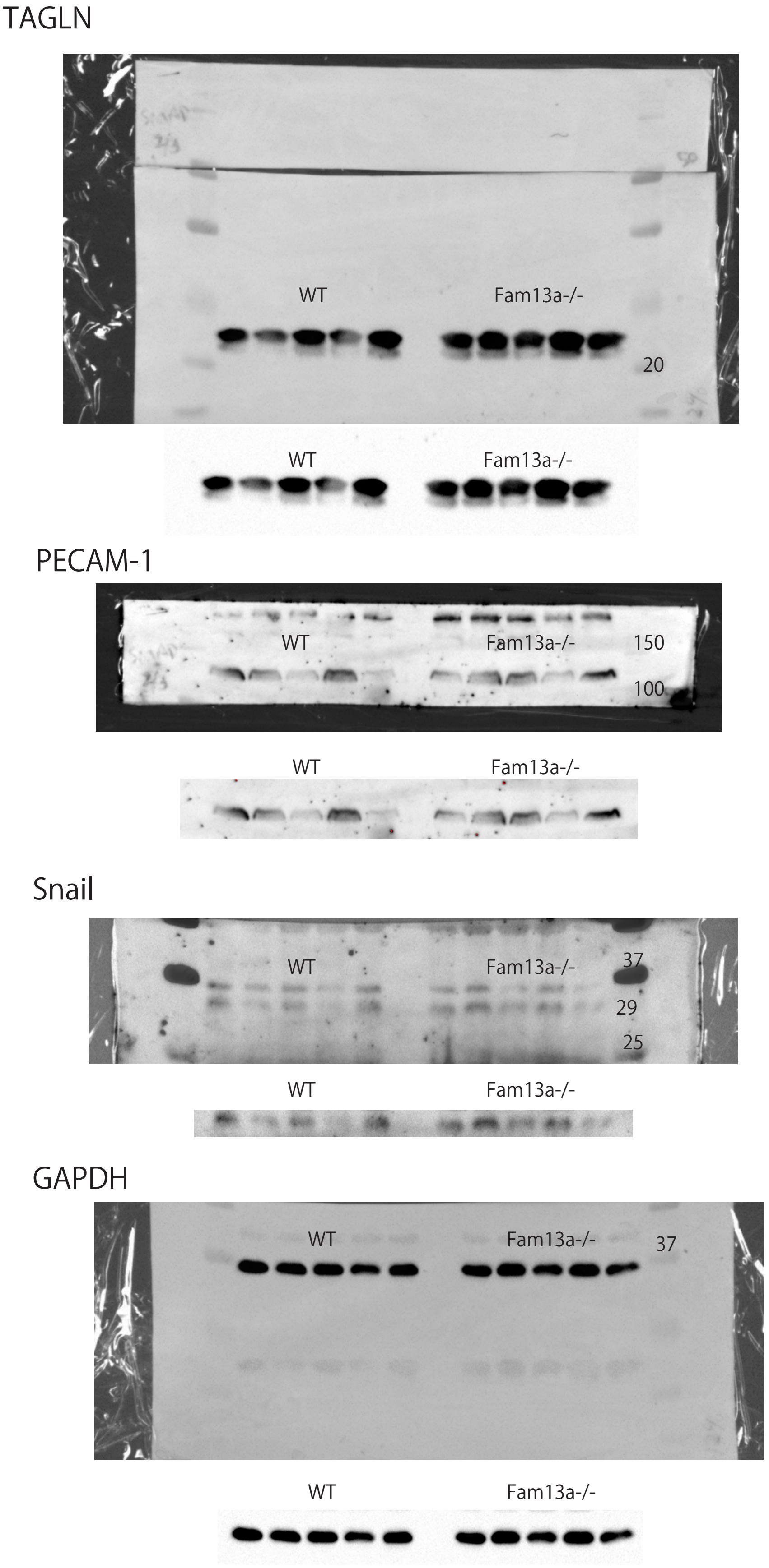

Samples: Mouse whole-lung

All blots were captured and processed using Chemi Doc XRS+ and Image Lab software

Figure 5H
